# Antidepressant drug switching in the Swiss population with a focus on Escitalopram and drugs with pharmacogenetic dosing guidelines: a drug utilization study using claims data

**DOI:** 10.1038/s41397-025-00382-1

**Published:** 2025-08-08

**Authors:** M. M. Roth, C. R. Meier, C. A. Huber, H. E. Meyer zu Schwabedissen, S. Allemann, C. Schneider

**Affiliations:** 1 Basel Pharmacoepidemiology Unit, Division of Clinical Pharmacy and Epidemiology, Department of Pharmaceutical Sciences, University of Basel, 4003 Basel, Switzerland; 2 Hospital Pharmacy, University Hospital Basel, 4056 Basel, Switzerland; 3 Department of Health Sciences, Helsana Insurance Group, 8001 Zürich, Switzerland; 4 Biopharmacy, Department of Pharmaceutical Sciences, University of Basel, 4003 Basel, Switzerland; 5 Pharmaceutical Care, Department of Pharmaceutical Sciences, University of Basel, 4003 Basel, Switzerland

**Keywords:** Risk factors, Genotype

## Abstract

Depression affects around 10% of the Swiss population. While SSRIs are commonly prescribed, only 30–40% of patients achieve remission. Pharmacogenetic (PGx) factors may explain part of this high rate of SSRI treatment failure. This study examined antidepressant (AD) switching among Swiss patients using escitalopram, focusing on whether they switched to ADs with PGx dosing guidelines (PGx AD) or ADs without PGx dosing guidelines (non-PGx ADs). Data from Swiss health insurance records identified 41 275 patients who used escitalopram between July 2020 and June 2022. While 6.4% (n = 2 638) switched to another antidepressant, only 35.4% of these opted for a PGx AD. Men, younger adults showed higher switching rates, whereas patients on antipsychotic medications switched less. Individuals younger than 20 years old and women were more likely to switch to PGx AD whereas the elderly were less likely to switch to PGx AD.

## Introduction

Depression is a mental health condition affecting approximately 10% of the Swiss population. The chronic and recurrent nature of depression necessitates effective long-term management [1]. Selective serotonin reuptake inhibitors (SSRIs) represent a fundamental element of the pharmacological management of major depressive disorder (MDD). In many guidelines, SSRIs are considered the first-line AD drug class, although some associations are moving towards patient individualization, such as the Association of Scientific Medical Societies (AWMF) [2–5]. However, treatment outcomes are often suboptimal, with 30–50% of patients not responding adequately to AD treatment. As a result, many patients experience adverse effects or an insufficient therapeutic response, leading clinicians to consider switching patients to alternative ADs [6–8]. A study conducted in the United Kingdom revealed that approximately 9.3% of patients initiating treatment with escitalopram required a switch to another AD [9].

One of the reasons for an inadequate response to escitalopram could be the genetic make-up of the patient. The metabolism of escitalopram and other SSRIs is catalysed by cytochrome P450 enzymes; particularly CYP2C19 and CYP2D6 are of relevance in the metabolism of SSRIs [10]. These enzymes exhibit significant inter-individual variability due to genetic polymorphisms, which can influence drug metabolism and, consequently, the efficacy and tolerability of substrate drugs [10, 11]. In accordance with this assumption, Jukić et al. found that patients categorized as poor metabolizers of CYP2C19 switched from escitalopram 3.3 times more frequently than those with normal metabolic capacity [12].

To the best of our knowledge, AD switching patterns on a population level have not yet been assessed in Switzerland. Not all ADs are affected by PGx and therefore do not have PGx dosing guidelines [10, 13–16]. The primary objective of this study was to examine the switching pattern of escitalopram to alternative ADs and to compare the rates of switching to PGx AD versus non-PGx ADs in the Swiss population. Rather than aiming to evaluate treatment effects or clinical decision-making at the individual level, the study sought to generate population-level insights into current prescribing practices in the Swiss healthcare context.

## Methods

### Study design and data source

We conducted a descriptive, retrospective study using claims data from Helsana Group, a Swiss health insurance company that covers approximately 15% of the Swiss population across all age groups. Helsana Group claims data include information on outpatient drug claims, categorized according to the World Health Organization’s Anatomical Therapeutic Chemical (ATC) classification system, as well as demographic information such as canton of residence, year of birth, and sex. However, clinical information, lifestyle factors such as smoking status and weight, use of over-the-counter medications, and treatment indications were not available in our anonymized dataset. The Helsana Group database has already been used in various drug safety and utilization studies [17–20].

### Study population

The study period ranged from July 1, 2020, to June 30, 2022. This study focused on escitalopram, the most frequently claimed AD in 2021. Our study population included all individuals with claims for escitalopram and at least one other medication for AD during the specified period.

### Antidepressants

We identified AD claims using the ATC codes N06AA (non-selective monoamine reuptake inhibitors or tricyclic ADs), N06AB (selective serotonin reuptake inhibitors), code), N06AF + N06AG (monoamine oxidase inhibitors (MAOIs)), and N06AX (other ADs) that subsume nonuniform underlying mechanisms of action (atypical ADs), such as serotonin-norepinephrine reuptake inhibitors (SNRIs) and tetracyclic ADs. We classified the respective AD as “pharmacogenetic” if any dosing guidelines were available in the Pharmacogenetic Knowledge Base (PharmGKB) in August 2024 [21]. The presence of a dosing guideline in PharmGKB corresponds to the highest evidence level (Level 1 A), which indicates variant-specific prescribing guidance in a current clinical guideline or FDA-approved drug label, supported by at least one additional peer-reviewed publication [22]. Based on this criterion, we identified dosing recommendations for the following 12 ADs: imipramine, clomipramine, trimipramine, amitriptyline, nortriptyline, doxepin, citalopram, paroxetine, sertraline, fluvoxamine, escitalopram, and venlafaxine. We excluded the lowest strengths for trazodone (50 mg) and mirtazapine (15 mg) because these are mostly prescribed for insomnia. Also, we excluded amitriptyline (10 mg) because it is mostly prescribed for neuropathic pain.

### Treatment change

We defined a potential switch in AD therapy as the co-occurrence of an escitalopram claim and a claim for another AD within a 100-day window. To identify switches, we analysed all AD claims for each patient. For each claims date of escitalopram, we checked whether a claim for a different AD was recorded within a 100-day period after the given date. This timeframe reflects the common practice in Switzerland of dispensing drugs for three-month periods, with an allowance for some potential non-adherence. If this co-occurred once during the study period, we checked if the next claim was again the new AD to classify whether it was a real switch or if it was a one-time prescription of another AD. We categorized cases with multiple overlaps during the 100-day window as combination therapies, except for cases in which switching to an AD required a combination of multiple strengths to achieve the correct dosage. We focused only on the first switch if more than one switch occurred during the study period.

### Drugs used for other conditions

Seven drug categories were identified using ATC codes: drugs used for cardiovascular diseases (B01AA, B01AC, C01, C04A, C02, C07, C08, and C09), diabetes mellitus (A10A, A10B, A10X), cancer (L01), epilepsy (N03), psychoses (N05A), pain (N02A, N02B), and respiratory illnesses (R03). We chose these categories because these drug classes are associated with side effects and interactions of ADs, and the indications for these drugs are associated with depression.

### Statistical analyses

We calculated the absolute and relative numbers of patients who switched from escitalopram to alternative ADs during the study period stratified by age, sex and region. Regional variables included the major regions of Switzerland, which are the reference areas defined by the Swiss Federal Office of Statistics (Zurich, Espace Mittelland, Lemanic region, Northwestern Switzerland, Eastern Switzerland, Ticino, and Central Switzerland) of the Swiss Confederation at the cantonal level. We ranked ADs according to the number of patients who switched to these ADs. Additionally, we calculated the number of patients switching each month and the proportion of patients switching to PGx AD or non-PGx AD.

For each drug category used for other conditions, we calculated the prevalence by dividing the number of individuals with at least one medication prescription in one of the defined ATC groups by the total number of patients during the study period. Age at the beginning of the study period was considered in all the calculations. We reported the frequencies and proportions as absolute numbers and percentages of samples.

We employed multivariable logistic regression to examine the factors associated with AD switching. Independent variables included in the regression model were age groups ( ≤ 19, 20–39, 40–64, 65–79, ≥80 years), sex (female or male), residence area (Zurich, Espace Mittelland, Lemanic region, Northwestern Switzerland, Eastern Switzerland, Ticino, Central Switzerland), claiming other drugs (yes/no), and the number of drug categories used for other conditions. We calculated the estimates as odds ratios (ORs) with 95% confidence intervals (CIs). To address the possibility of overfitting or chance findings, we performed a sensitivity analysis in which we re-estimated the logistic regression model including only age group, sex, and the use of antipsychotic medication. We extrapolated our results to the Swiss population in Appendix Tables 1 and 2. The extrapolation factor (ef) is based on a person’s age, gender, and canton of residence. It is provided annually and individually by the joint facility KVG (‘Krankenversicherungsgesetz’). The ef is used for risk balancing among Swiss mandatory basic health insurances and is therefore based on the total number of insured persons of all Swiss insurance companies [23]. All statistical analyses were performed using SAS (version 9.4; SAS Institute Inc., Cary, NC, USA). In accordance with the Swiss Federal Law on Data Protection (Article 22), ethics approval was not required, as the study was retrospective and the data were anonymized [24]. Statistical significance was set at P < 0.05.

### Large language models

We used Deepl Write (Deepl SE, Germany) for finale language editing.

## Results

### Description of the study population

We identified 41 275 individuals who claimed at least one prescription for escitalopram during the study period. Of those 16 581 individuals aged 4 −103 years (mean 56.0 year ± 20.8 years) claimed at least one prescription for escitalopram and another AD during the study period, qualifying them as potential switchers. The study population comprised 66.7% women. The majority of the study population was 40 years or older with the largest age group being 40–64 years (39.9%). More than 95% of the study population had in addition to the prescribed AD at least one prescription for drugs from another drug class, the top 3 being drugs for pain, cardiovascular drugs, and drugs to treat psychoses. Every 7^th^ person (15.9%) in our study population (i.e. people who claimed escitalopram and at least one other medication for AD) switched from escitalopram to another AD during the study period. In relation to all escitalopram users - irrespective of whether or not they also claimed another AD drug - 6.4% switched. Details on the characteristics of the study population stratified by escitalopram switching status are provided in Table 1 and Appendix Table 1.

**Table 1:** Characteristics of the study population.

Characteristics of the study population		
	Without switches	With switches
	[N, %]	[N, %]
Total	13 943, 84.1	2 638, 15.9
Gender		
Male	4 567, 32.8	953, 36.1
Female	9 376, 67.2	1 685, 63.9
Age mean (SD)	56.2 (20.8)	54.8 (20.8)
Age group (in years)		
0–19	338, 2.4	64, 2.4
20–39	3 054, 21.9	652, 24.7
40–64	5 584, 40.1	1 033, 39.2
65–79	2 474, 17.7	472, 17.9
80+	2 493, 17.9	417, 15.8
Region		
Zurich	3 213, 23.0	563, 21.3
Espace Mittelland	3 103, 22.3	598, 22.7
Lemanic Region	2 169, 15.6	435, 16.5
Northwestern Switzerland	1 779, 12.8	404, 15.3
Eastern Switzerland	1 538, 11.0	279, 10.6
Ticino	1 061, 7.6	187, 7.1
Central Switzerland	1 009, 7.2	158, 6.0
Missing Information about region	71, 0.5	14, 0.5
Number of other drug classes		
none	292, 2.1	70, 2.7
1–2	1 246, 8.9	260, 9.9
3–4	3 126, 22.4	667, 25.3
≥5	9 279, 66.6	1 641, 62.2
Other drug classes		
Drugs for cardiovascular diseases	11 748, 84.3	2 177, 82.6
Drugs for diabetes mellitus	1 833, 13.1	306, 11.6
Drugs for cancer	838, 6.0	163, 6.2
Drugs for epilepsy	1 293, 9.3	232, 8.8
Drugs for psychoses	5 289, 37.9	912, 34.7
Drugs for pain	11 148, 79.9	2 059, 78.1
Drugs for respiratory illness	3 523, 25.3	912, 34.6

*N* number of persons; *%* percentage of all switchers or non-switchers present during the study period; *SD* standard deviation.

### Switching behaviour

**Fig. 1:**
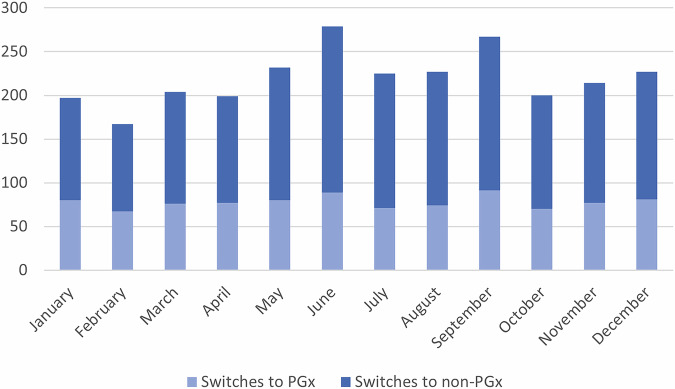
Number of patients on escitalopram switching to another PGx or non-PGx AD per month. Fehler! Textmarke nicht definiert.

Among those who switched, 15.9% switched to another SSRI, 10.0% to tricyclic AD (TCA), and 74.1% switched to other ADs. Most switches (61.6%) occurred to Mirtazapine, Duloxetine, Venlafaxine, Trimipramine and Amitriptyline. Only 35.4% of patients switched to another PGx AD. In 77.9% of the patients who switched to another SSRI, the patient switched to a PGx SSRI. No switches to PGx TCA were observed. While men were more likely to switch to another AD in general, women were more likely to switch to a PGx AD, yielding an OR of 1.16 (95% CI 1.06–1.26) and of 0.78 (95% CI 0.65, 0.93), respectively. People with a higher number of concomitant medications were more likely to switch to a PGx AD than people without (OR 1.75, 95% CI 0.83–3.82), although results were not statistically significant. Concomitant medication was not associated with switching in general (see Tables 2 and 3 and Appendix Tables 2 for the detailed switching characteristics). The sensitivity analysis showed that the direction and statistical significance of the primary associations remained unchanged, suggesting that the results are robust to model simplification. The number of patients who switched during the year ranged from 167 to 279 per month, with the fewest switches observed in February and the most in June. Figure 1 illustrates the monthly distribution of patient switches throughout the year

**Table 2:** Characteristics of the patient population switching from Escitalopram to another AD.

	Total [N, %]		Switched to TCA, [N, %]		Switched to SSRI, [N, %]		Switched to other AD, [N, %]	
	2 638		263, 10.0		420, 15.9		1 955, 74.1	
	nPGx	PGx	nPGx	PGx	nPGx	PGx	nPGx	PGx
Total	1 705, 64.6	933, 35.4	0, 0.0	263, 28.2	93, 5.5	327, 35.0	1 612, 94.5	343, 36.8
Gender								
Male	647, 37.9	306, 32.8	0, 0.0	79, 30.0	24, 25.8	103, 31.5	623, 38.6	124, 36.2
Female	1 058, 62.1	627, 67.2	0, 0.0	184, 70	69, 74.2	224, 68.5	989, 61.4	219, 63.8
age group (in years)								
0–19	34, 2.0	30, 3.2	0, 0.0	10, 3.8	3, 3.2	13, 4.0	31, 1.9	7, 2.1
20–39	399, 23.4	253, 27.1	0, 0.0	40, 15.2	36, 38.7	93, 28.5	363, 22.5	12, 35.0
40–64	652, 38.2	381, 40.8	0, 0.0	115, 43.7	45, 48.4	126, 38.5	606, 37.6	140, 40.8
65–79	309, 18.1	163, 17.5	0, 0.0	66, 25.1	5, 5.4	53, 16.2	304, 18.9	44, 12.8
80+	311, 18.3	106, 11.4	0, 0.0	32, 12.2	4, 4.3	42, 12.8	307, 19.1	32, 9.3
Region								
Zurich	360, 21.1	203, 21.8	0, 0.0	60, 22.8	14, 18.3	73, 22.3	343, 21.3	70, 20.4
Espace Mittelland	393, 23.0	205, 21.9	0, 0.0	60, 22.8	19, 20.5	74, 22.6	373, 23.2	71, 20.7
Lemanic Region	254, 14.9	180, 19.3	0, 0.0	20, 7.6	32, 34.4	78, 23.9	222, 13.8	82, 23.9
Northwestern Switzerland	278, 16.3	126, 13.5	0, 0.0	42, 16.0	3, 3.2	34, 10.4	274, 17.0	50, 14.6
Eastern Switzerland	182, 10.7	97, 10.4	0, 0.0	44, 16.7	4, 4.3	24, 7.3	178, 11.1	29, 8.5
Ticino	122, 7.2	66, 7.1	0, 0.0	12, 4.6	10, 10.7	32, 9.8	112, 6.9	22, 6.4
Central Switzerland	105, 6.2	53, 5.7	0, 0.0	25, 9.5	7, 7.5	11, 3.4	98, 6.1	17, 4.9
No information about region	11, 0.6	3, 0.3	0, 0.0	0, 0.0	1, 1.1	1, 0.3	10, 0.6	2, 0.6

*AD* antidepressant; *N* number of persons; *%* percentage of the corresponding total in the column, *PGx* pharmacogenetic dosing guidelines available; *nPGx* pharmacogenetic dosing guidelines not available.

**Table 3:** Association between sociodemographic factors or comedication and AD switching in patients on treatment with escitalopram.

Variables	Switch		Switch to PGx AD	
	OR	P	OR	P
Age group^a^				
0–19	0.98 (0.74–1.30)	0.94	1.66 (0.98–2.80)	0.06
20–39	1.14 (1.02–1.27)	0.02	1.13 (0.92–1.39)	0.25
40–64	Ref		Ref	
65–79	1.08 (0.95–1.22)	0.23	0.88 (0.69–1.11)	0.26
80+	0.97 (0.85–1.10)	0.62	0.54 (0.41–0.70)	<0.01
Sex				
Female	Ref		Ref	
Male	1.16 (1.06 −1.26)	<0.01	0.78 (0.65–0.93)	<0.01
Region^b^				
Zurich	Ref		Ref	
Espace Mittelland	1.09 (0.96–1.24)	0.17	0.92 (0.72–1.18)	0.52
Lemanic Region	1.12 (0.98–1.29)	0.09	1.23 (0.95–1.60)	0.12
Northwestern Switzerland	1.30 (1.13–1.50)	<0.01	0.80 (0.61–1.05)	0.11
Eastern Switzerland	1.03 (0.88–1.20)	0.72	0.98 (0.72–1.32)	0.87
Ticino	1.04 (0.87–1.25)	0.64	1.09 (0.76–1.55)	0.64
Central Switzerland	0.88 (0.72–1.06)	0.18	0.88 (0.60–1.28)	0.5
Number of other drug classes^c^				
1 or 2	0.99 (0.72–1.40)	0.98	1.32 (0.68–2.68)	0.42
3 or 4	1.06 (0.75–1.51)	0.74	1.69 (0.85–3.51)	0.15
≥5	0.94 (0.65–1.38)	0.76	1.75 (0.83–3.82)	0.15
Other drugs classes^c^				
Drugs for cardiovascular diseases	0.98 (0.85–1.14)	0.83	1.16 (0.87–1.55)	0.31
Drugs for diabetes mellitus	0.91 (0.78–1.04)	0.15	1.06 (0.82–1.38)	0.64
Drugs for cancer	0.98 (0.85–1.14)	0.37	0.86 (0.61–1.21)	0.4
Drugs for epilepsy	0.99 (0.85–1.15)	0.91	0.97 (0.72–1.30)	0.84
Drugs for psychoses	0.86 (0.81–0.97)	0.01	0.98 (0.81–1.17)	0.78
Drugs for pain	0.99 (0.87–1.13)	0.89	0.88 (0.68–1.12)	0.29
Drugs for respiratory illness	0.98 (0.89–1.09)	0.73	0.95 (0.78–1.16)	0.64

^a^age group 40–64 is the reference group; ^b^region Zurich is the reference group; ^c^no other drug classes is the reference group.

*PGx AD* antidepressant with pharmacogenetic dosing guideline; *P* P-Value; *Ref* Reference group; *OR* odds ratio; *CI* confidence interval.

## Discussion

In this study, we assessed the percentage of people switching from escitalopram to other ADs in the Swiss population. Based on extrapolations from the Helsana Group population in this study, which included 15% of the Swiss population, we estimated that 15.8% of the Swiss population who claimed escitalopram and at least one additional AD switched during 2021. In relation to all escitalopram users, 6.4% switched. Based on our analysis most patients switched from escitalopram to non-PGx ADs. The factors associated with switching were male sex, living in northwest Switzerland, and age between 20 and 39 years. Women were more likely to switch to PGx ADs than men were.

Our findings align with those of previous studies, which reported similar switching behaviors and rates among escitalopram users [9, 25–27]. The majority of switches from SSRIs were to other AD classes than SSRIs, a trend also noted in other studies, where venlafaxine and other non-SSRI agents were common alternatives following treatment failure [27–29]. Like in other studies, patients in our study most commonly switched from escitalopram to mirtazapine, duloxetine and venlafaxine [9, 28]. In our study, we observed no switch from escitalopram to non-PGx TCAs, possibly because non-PGx TCAs were rarely prescribed. Our study population exhibited demographic characteristics similar to those observed in other studies on antidepressant use, including a higher prevalence among women and older adults, as previously shown in a study using Helsana Group data [30].

Comparing the factors associated with switching in our study to those in other studies, we found that men and younger adults (aged 20–39 years), which partially mirrors findings from similar studies that also reported higher odds for switching among younger adults [9, 27, 29, 31]. We also observed a higher propensity of women to switch to PGx ADs, whereas older adults tend to switch less to PGx ADs, which has both not been assessed in previous studies [9, 27, 29, 31, 32].

Regarding pharmacogenetics, CYP2C19 slow and ultrarapid metabolizers switch ADs more frequently than do normal metabolizers [12, 32, 33]. In Switzerland, the prevalence of poor and (ultra)rapid metabolizers is 3% and 30%, respectively [34]. We did not have information on the genotype of the registered persons and were unable to assess whether ultrarapid or slow metabolizers were more prevalent among switchers. Whether the genetic predisposition of CYP2C19 affects AD switching remains unclear, as a meta-analysis showed that some studies found no impact of metabolic phenotypes on escitalopram efficacy [35]. While some of the observed switches may align with pharmacogenetically informed decisions, it is equally likely that they reflect prescribers’ adherence to clinical guidelines, patient tolerability, or institutional prescribing habits. These inconsistencies, due to methodological differences, highlight the need for further research on PGx-guided AD prescription and its real-world effectiveness.

A key strength of our study is the use of a large health insurance claims database that captures data from approximately 15% of the total Swiss population, thereby providing a substantial level of representativeness. Another strength is the inclusion of various sources for PGx recommendations to allow a comprehensive analysis, while restricting the analysis to drugs with PGx guidelines to ensure that they are relevant for current clinical decision-making. Our analysis relies on claims data and is subject to the limitations inherent in this type of data. The main limitation of this study was that the claims data did not provide genetic information or information about drug efficacy or tolerance. As a result, the reason for switching ADs remains unknown. Additionally, we had no information regarding OTC use or drug administration in hospitals. Therefore, the results for drugs that are also available as OTC drugs, as well as drugs prescribed in primary care but typically used in hospitals, will be underestimated. Regarding ADs, this should not be a major limitation because most depressed individuals are treated as outpatients and only hypericum herba is available OTC [36]. In Switzerland, there is a certain (small) number of out-of-pocket payments on an annual basis before health care insurance reimburses the bills. As out-of-pocket payments affect acute medication more than chronic medication, we may have missed a small number of drug claims, leading to a slight underestimation of acute medication. Additionally, the Helsana Group database does not provide information about the indication of the claimed ADs; therefore, we cannot ascertain whether ADs were indeed used for depression or other conditions.

## Conclusions

To the best of our knowledge, this is the first study to assess the patterns of AD switching including PGx factors using Swiss health care claims data at a population level. We found a switching rate of 6.4%, mostly from escitalopram to a non-PGx AD. To gain more insight into the influence of PGx on AD switching, further studies including genetic information are needed.

## Supplementary information

Appendix Table 1

Appendix Table 2

## Data Availability

The datasets analysed and/or generated during the current study are not available for the public due to confidentiality requirements issued by Helsana Group. Datasets and analysis codes can be made available by the corresponding author (s.allemann@unibas.ch) upon reasonable request and with permission of Helsana Group.
